# Neuroblastomas with chromosome 11q loss and single copy MYCN comprise a biologically distinct group of tumours with adverse prognosis

**DOI:** 10.1054/bjoc.2001.1960

**Published:** 2001-08

**Authors:** M E M Oude Luttikhuis, J E Powell, S A Rees, T Genus, S Chughtai, P Ramani, J R Mann, C M McConville

**Affiliations:** Division of Medical and Molecular Genetics; Department of Public Health & Epidemiology, University of Birmingham, B15 2TT; Departments of Histopathology and; Haematology/Oncology, Birmingham Children's Hospital, Birmingham, B4 6NH, UK

**Keywords:** neuroblastoma, LOH, chromosome 11, MYCN

## Abstract

Neuroblastoma is a heterogeneous tumour and its effective clinical management is dependent on accurate prognostic evaluation. In approximately 25% of patients amplification of the MYCN oncogene is known to be associated with a poor outcome. In order to identify additional molecular markers with prognostic potential in non-MYCN-amplified neuroblastomas, we looked for a correlation between clinical outcome and loss of heterozygosity (LOH) on four chromosomes that frequently show alteration in neuroblastoma (chromosomes 3, 4, 11 and 14). Chromosome 11q loss (with frequent parallel loss of chromosomes 3p, 4p and/or 14q) was found exclusively in tumours without MYCN amplification and was significantly associated with poor event-free survival. The 2-year event-free survival rate for 11q LOH cases was 30%, compared to 34% for MYCN-amplified cases and 100% for cases without these abnormalities. While 11q LOH was associated predominantly with advanced-stage disease, 2 cases with low-stage disease and 11q LOH both suffered relapses. We conclude that chromosome 11q loss defines a biologically distinct group of tumours without MYCN amplification that appear to have potential for aggressive metastatic growth. Thus this genetic alteration may be an important new prognostic marker in neuroblastoma. © 2001 Cancer Research Campaign http://www.bjcancer.com


British Journal of Cancer (2001) 85(4), 531–537
© 2001 Cancer Research Campaign
doi: 10.1054/ bjoc.2001.1960, available online at http://www.idealibrary.com on IDEAL

http://www.bjcancer.com

### Neuroblastomas with chromosome 11q loss and single copy MYCN comprise a biologically distinct group of tumours with adverse prognosis

**MEM Oude Luttikhuis[1], JE Powell[2], SA Rees[1], T Genus[1], S Chughtai[1], P Ramani[3], JR Mann[4] and CM McConville[1]**

[1]Division of Medical and Molecular Genetics, and [2]Department of Public Health & Epidemiology, University of Birmingham B15 2TT, [3]Departments of Histopathology and [4]Haematology/Oncology, Birmingham Children's Hospital, Birmingham, B4 6NH, UK

**Summary** Neuroblastoma is a heterogeneous tumour and its effective clinical management is dependent on accurate prognostic evaluation. In approximately 25% of patients amplification of the MYCN oncogene is known to be associated with a poor outcome. In order to identify additional molecular markers with prognostic potential in non-MYCN-amplified neuroblastomas, we looked for a correlation between clinical outcome and loss of heterozygosity (LOH) on four chromosomes that frequently show alteration in neuroblastoma (chromosomes 3, 4, 11 and 14). Chromosome 11q loss (with frequent parallel loss of chromosomes 3p, 4p and/or 14q) was found exclusively in tumours without MYCN amplification and was significantly associated with poor event-free survival. The 2-year event-free survival rate for 11q LOH cases was 30%, compared to 34% for MYCN-amplified cases and 100% for cases without these abnormalities. While 11q LOH was associated predominantly with advanced-stage disease, 2 cases with low-stage disease and 11q LOH both suffered relapses. We conclude that chromosome 11q loss defines a biologically distinct group of tumours without MYCN amplification that appear to have potential for aggressive metastatic growth. Thus this genetic alteration may be an important new prognostic marker in neuroblastoma. © 2001 Cancer Research Campaign http://www.bjcancer.com

**Keywords**: neuroblastoma, LOH, chromosome 11, MYCN

Neuroblastoma is a tumour of the neural crest derived sympathetic nervous system, which in the UK accounts for 13% of paediatric cancer deaths (Powell et al, 1998). The clinical behaviour of the tumour varies widely, and although the International Neuroblastoma Staging System (INSS) (Brodeur et al, 1993), which attempts to classify the extent of disease based on clinical and pathological characteristics, provides a useful guide to prognosis, nevertheless tumour behaviour is still unpredictable in some cases. Thus, patients with localized disease (INSS stages 1 and 2) generally have excellent survival rates, but a proportion subsequently relapse and die of their disease. Similarly, the regression or resolution, frequently cited as a characteristic feature of stage 4S tumours in infants, is not always seen, and progressive fatal tumour growth may also occur (Kerbl et al, 1996). In addition, although widespread disease (stages 3 and 4) is very frequently associated with an extremely poor prognosis, the pattern of disease progression may be rapid, causing early death, or may follow a more protracted course over several years. A small proportion of stage 4 patients may be long-term survivors.

The variability in tumour behaviour has led to the suggestion that neuroblastoma comprises a heterogeneous group of developmentally related neoplasms (Brodeur et al, 1997) that may be assumed to have differing genetic aetiologies. The genetic defect(s) underlying the development of neuroblastoma are not well understood however, and the significance of the large number of different chromosomal abnormalities seen in tumours is unclear. Currently the only clinically useful genetic marker is amplification of the MYCN oncogene. Amplification is seen in 20–30% of primary untreated tumours (Bown et al, 1999; Guo et al, 1999), and is very frequently associated with a poor outcome.

More recent cytogenetic and molecular studies have identified a number of additional recurrent chromosomal abnormalities of varying frequency and prognostic significance. Deletion of chromosome 1p occurs in approximately 30% of tumours (Caron, 1995; Fong et al, 1992) and is often observed in combination with MYCN amplification. Chromosome 17q gain is observed in approximately half of all tumours, and is associated with a low survival rate (Bown et al, 1999), although this risk appears to be modified by other factors such as MYCN amplification and chromosome 1p deletion. Loss of heterozgyosity (LOH) and comparative genomic hybridization (CGH) studies have implicated chromosomes 3, 4, 11 and 14 (Brinkschmidt et al, 1998; Caron et al, 1996; Ejeskar et al, 1998; Lastowska et al, 1997; Plantaz et al, 1997; Vandesompele et al, 1998), but with the exception of chromosome 11 loss, which is most frequently observed in advanced-stage tumours, prognostic significance is uncertain. There is some evidence to suggest however, that loss on these 4 chromosomes is related and occurs in a defined subgroup of non-MYCN amplified neuroblastomas (Bown et al, 1999; Guo et al, 1999; Plantaz et al, 1997).

In this study we have carried out for the first time a detailed investigation of the clinical significance of LOH on chromosomes 3, 4, 11 and 14 in a single patient group. We present evidence that chromosome 11q LOH is highly predictive of disease progression, irrespective of clinical stage. Furthermore our results are strongly suggestive of the existence of 2 distinct subgroups of neuroblastoma,

*Received 1 December 2000*
*Revised 13 May 2001*
*Accepted 15 May 2001*

*Correspondence to:* CM McConville both with poor prognosis but with different genetic aetiologies, i.e. amplification of MYCN or chromosome 11q loss.

### PATIENTS AND METHODS

### Patients

Tumour and matched normal tissue samples were collected from 28 patients presenting at the Birmingham Children's Hospital (UK) between 1992 and 1999 (out of a total of 66 patients treated at the hospital during that period). Patients were selected for study based on the availability of tumour material. There were 23 neuroblastomas, 4 ganglioneuroblastomas and 1 ganglioneuroma. Tumour staging was carried out according to the International Neuroblastoma Staging System criteria (Brodeur et al, 1993). The details of all patients are summarized in Table 1. Tumour samples were obtained prior to treatment in 23 cases, at relapse in 2, and for 1 ganglioneuroblastoma, the sample was obtained post-chemotherapy. Early passage cell lines, established from 2 stage 4 tumours (including 1 diagnostic sample obtained prior to treatment), were also used in this study.

All patients were treated using standard European neuroblastoma protocols: LENSG 1994 for localized tumours in patients > 1 year, ENSG 8 for neuroblastoma in infants, ENSG 5 for stage 4 and MYCN-amplified tumours and ENSG 9 for stage 2B/3 tumours. With the exception of localized and stage 4S tumours, all protocols include a chemotherapy regimen involving alternating courses of vincristine 1.5 mg/m$^2$, cisplatin 80 mg/m$^2$, etoposide 200 mg/m$^2$, and cyclophosphamide 600 mg/m$^2$ (OPEC) and vincristine, carboplatin 500 mg/m$^2$, etoposide and cyclophosphamide (OJEC) prior to surgery. Additionally in the case of ENSG5, surgery is followed by high-dose melphalan and autologous bone marrow or peripheral stem cell rescue. The median follow-up time of the series was 29 months (range 4–73 months). The study was approved by the regional Research Ethics Committee and samples were obtained with appropriate informed consent.

### LOH analysis

DNA was extracted from tumours and corresponding normal tissue (blood or lymphoblastoid cell lines) according to standard methods. Tumours were selected on the basis of availability of material. The pathology report for each sample was checked to ensure that tumour cells were contained within the tissue. Although each tissue sample may vary in tumour content from one area to another, nevertheless the fact that all tumours were collected from the same local centre did allow close liason with the pathologist as to the requirements for molecular studies and ensured that tissue from the centre of the specimen rather than the margins was collected.

Polymorphic markers from chromosomal regions 3p, 4p, 11q and 14q were used to screen for LOH: D3S4550 3p24, D3S1767 3p22, D3S2408 3p21; D4S432, MSX1, D4S2375, D4S2366, all

**Table 1** Clinical and genetic characteristics of neuroblastoma patients

| Patient No. | Stage | Sex | Age at diagnosis (years–months) | Tumour histology* | Outcome† | NMA‡ | 1p36 loss§ | Chromosome | | | |
|---|---|---|---|---|---|---|---|---|---|---|---|
| | | | | | | | | 3p | 4p | 11q | 14q¶ |
| 11 | 1 | m | 0–4 | FH | CR | – | – | – | – | – | – |
| 4 | 1 | m | 0–10 | FH | CR | – | – | – | – | – | – |
| 13 | 1 | f | 1–0 | FH | CR | – | – | – | – | – | – |
| 8 | 1 | m | 1–2 | FH | CR | – | – | – | – | – | – |
| 10 | 1 | f | 2–2 | FH | CR | – | – | – | – | – | – |
| 27 | 1 | m | 4–5 | FH | CR | – | – | – | – | – | – |
| 26 | 1 | m | 5–10 | UH | CR2 | – | – | – | – | LOH | – |
| 24 | 1 | f | 7–5 | FH | CR | – | – | – | – | – | – |
| 34 | 2B | m | 1–4 | FH | CR | – | – | – | – | – | – |
| 30 | 2B | m | 4–11 | UH | AWD | – | – | – | LOH | LOH | LOH |
| 5 | 4S | f | 0–2 | FH | CR | – | – | n.i. | – | – | – |
| 21 | 3 | m | 0–10 | UH | CR | – | n.d. | – | – | – | – |
| 18 | 3 | f | 1–0 | FH | CR | – | + | – | LOH | LOH | LOH |
| 23 | 4 | m | 2–4 | UH | AWD | – | – | LOH | – | LOH | – |
| 28 | 4 | m | 2–6 | n.d. | DOD | – | + | LOH | LOH | LOH | – |
| 6 | 4 | m | 3–0 | UH | CR | – | – | LOH | LOH | LOH | – |
| 15 | 4 | m | 3–2 | UH | DOD | – | – | LOH | – | LOH | n.i. |
| 17 | 4 | m | 3–3 | FH | DOD | – | + | LOH | LOH | LOH | LOH |
| 7 | 4 | m | 3–8 | UH | DOD | – | – | – | – | LOH | – |
| 14 | 4 | m | 3–9 | UH | DOD | – | + | – | LOH | LOH | – |
| 1 | 4 | m | 7–10 | UH | DOD | – | – | LOH | LOH | – | – |
| 29** | 4S | m | 0–1 | FH | DOD | + | + | – | – | – | – |
| 36** | 4S | m | 0–5 | FH | CR | + | – | – | – | – | – |
| 20 | 3 | m | 0–9 | UH | CR | + | – | – | – | – | – |
| 22 | 4 | m | 0–10 | UH | DOD | + | + | – | – | – | – |
| 16 | 4 | f | 1–1 | UH | DOD | + | – | – | – | – | – |
| 31 | 4 | f | 2–6 | UH | CR | + | + | – | – | – | – |
| 32 | 4 | m | 4–9 | UH | AWD | + | + | – | LOH | – | – |

*FH/UH: favourable/unfavourable histology (Shimada); †CR: complete remission; CR2: 2nd remission; AWD: alive with disease; DOD: dead from disease; ‡NMA: +: MYCN amplified, –: MYCN single copy; §+: 1p36 loss, –: 1p36 retention; ¶LOH: loss of heterozygosity; n.i.: not informative; –: retention of heterozygosity; **patients 29 & 36 were stage 4S by INSS criteria, but were treated as stage 4 because of MYCN amplification and disease progression.

 *© 2001 Cancer Research Campaign*

4p16; D11S2632 11q21, D11S1366 11q22, D11S1998 11q23, D11S4464 11q24; D14S617, D14S1426, D14S118, all 14q32. Data on the chromosomal localization of polymorphic markers were extracted from the United Database for Human Genome Mapping (http://bioinformatics.weizmann.ac.il/udb) (Chalifa–Caspi et al, 1998). PCR products amplified from each normal/tumour pair were run in adjacant lanes on 6% denaturing polyacrylamide gels and were visualized by silver staining. LOH was scored where at least one marker was informative and showed ≥ 60% reduction in allele intensity as determined by densitometry.

### MYCN and chromosome 1p analysis

MYCN status was determined by FISH using standard methods. Tumours were considered to have amplified MYCN when copy number was increased > 5-fold. Chromosome 1p status was determined by LOH, and in most cases, also by FISH.

### Statistical analysis

The Fisher's exact test was used to examine associations between LOH and stage, age, gender and MYCN status. The Kaplan–Meier method and the log rank test were used to estimate overall and event-free actuarial survival. Event-free survival was defined as the time between diagnosis and the first clinical signs of disease progression, or death without disease progression. In order to determine the representativeness of the patient sample studied, chi-square tests were used to compare the characteristics of cases used in the study with those diagnosed in the same period, but for whom material was unavailable.

### RESULTS

### LOH analysis

We studied 28 paired samples of normal and tumour tissue for LOH using markers mapping within recurrent regions of loss on chromosomes 3p21–24, 4p16, 11q14–23 and 14q32 respectively. MYCN amplification and chromosome 1p36 status were also determined for all tumours (Table 1). The observed frequency of LOH in the tumour panel was 22% on chromosome 3 (6 out of 27 informative samples), 29% on chromosome 4 (8 out of 28), 36% on chromosome 11 (10 out of 28) and 11% on chromosome 14 (3 out of 27). Although the frequency of LOH was not high, nevertheless the pattern of loss was striking in two respects, firstly there was a high degree of concordance between LOH on each of these 4 chromosomes: out of 10 tumours with chromosome 11q LOH, 5 also had LOH on at least 2 other chromosomes (Table 1). In addition it was noted that there was an almost complete absence of LOH in MYCN-amplified tumours and a significant negative association between 11q LOH and MYCN amplification ($P$ = 0.03). These observations suggest that neuroblastomas with MYCN amplification and those with LOH form 2 distinct groups in which the molecular events associated with tumourigenesis differ. In the latter group, LOH on chromosome 11q is most frequent and appears to be the most critical event, although the recurrent parallel loss on chromosomes 3, 4, and 14 suggests that these are also significant alterations.

There was no clear association between loss of chromosome 1p36 and LOH on other chromosomes. 1p36 loss was observed in 30% of tumours overall, with an approximately equal frequency in MYCN-amplified tumours and the group of tumours showing LOH on chromosomes 3, 4, 11 and 14.

### Prognostic significance of chromosome 11q LOH

When considering the possible prognostic significance of the observed genetic alterations, in view of the relatively small size of the study it was important to ensure that our study population was representative of neuroblastoma patients generally. In order to exclude the possibility of sample bias, a comparison was made between the clinical features of the patients studied with the features of a further group of patients ($n$ = 38) presenting during the same time period, but not studied due to unavailability of material.

No significant differences were observed between the 2 groups (cases studied vs cases not included) with respect to age (29% were under 1 year of age in both groups) or gender (25% vs 34% female, $P$ = 0.59). We noted however, that high-stage tumours were under-represented in the study sample, and low-stage tumours were over-represented (due to the greater availability of the latter, which are totally resected without chemotherapy). However, after stratifying for low- and high-stage disease, no significant difference was found in the overall survival rate of the 2 groups (2-year survival: 64% vs 66%, stratified log rank test, $P$ = 0.93). We conclude therefore that, while low-stage patients are over-represented in our sample, in terms of survival and other prognostic factors, the group is typical of neuroblastoma patients in general and sample bias is unlikely to have had a significant influence on the associations observed.

Among patients with single-copy MYCN, the occurrence of chromosome 11q LOH was associated with advanced-stage disease ($P$ = 0.009) and also a reduction in both event-free ($P$ = 0.0003) and overall survival ($P$ = 0.006) as determined by Kaplan–Meier analysis and a log rank test (Figure 1). The 2-year event-free survival rate was only 30%, compared with 100% among patients without 11q LOH. Similarly the overall survival was reduced from 100% to 45%. Although the significance of these observations must be treated with caution because of the small sample size ($n$ = 21), nevertheless the occurrence during the total period of the study, of 5 deaths and 3 relapses among the 11q LOH group ($n$ = 10), in contrast to only 1 death among 11 patients without 11q LOH is strongly suggestive of an important role for genes on chromosome 11q in tumour progression/metastasis.

It is of interest that although 11q LOH was associated predominantly with advanced-stage neuroblastoma, 2 patients with low-stage tumours that showed 11q LOH, subsequently relapsed (patients 26 and 30, Table 1). Both patients presented with similar clinical features: age approximately 5–6 years at diagnosis, a cervical/thoracic tumour (stage 1 or 2) with unfavourable histology, and absence of MYCN amplification. Metastatic disease was absent or was confined to local ipsilateral lymph nodes. Disease remission was obtained in both cases following treatment (surgery ± chemotherapy), but both patients subsequently relapsed with either local (patient 30) or widespread disease (patient 26). These observations suggest that 11q LOH may potentially provide a very useful prognostic marker to identify a subgroup of patients who have low-stage tumours but who are at high risk of relapse.

The prognostic significance of LOH on chromosomes 3, 4 and 14 is more difficult to assess in view of the smaller number of patients involved, and the very frequent co-occurrence with 11q LOH. Only 2 patients showed LOH on chromosomes 3, 4 or 14 in

© 2001 Cancer Research Campaign

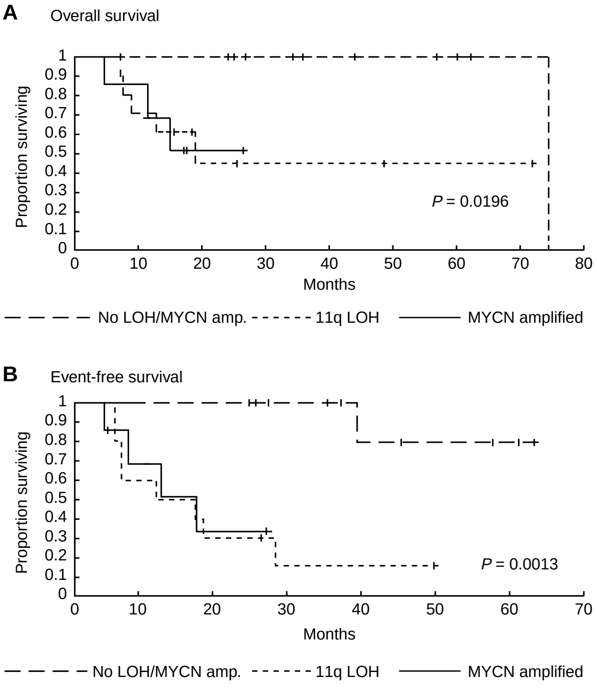

**Figure 1** Overall survival (**A**) and event-free survival (**B**) for neuroblastoma patients with MYCN amplification ($n$ = 7), with 11q LOH ($n$ = 10) or without molecular abnormalities ($n$ = 11). $P$-values for OS and EFS are 0.0196 and 0.0013 respectively for comparison of equality of survival distribution for all 3 patient groups, and 0.0057 (OS) and 0.0003 (EFS) for comparison of patients with 11q LOH and those without molecular abnormalities the absence of chromosome 11q LOH. 1 patient with a MYCN-amplified tumour showed chromosome 4 LOH. The second patient (patient no. 1 in Table 1) showed LOH on both chromosomes 3 and 4. This boy had an atypical disease presentation, with diagnosis at approximately 8 years of age, of a stage 4 adrenal neuroblastoma with multiple lymph node and bony metastases, but without bone marrow involvement. MYCN was not amplified. Despite intensive treatment, several subsequent recurrences in lymph nodes and bone eventually led to the patient's death approximately 6 years later. The final tumour specimen, which was still negative for MYCN amplification, was obtained approximately 3 months before the patient's death and was used for LOH studies. It is of interest that this is the only patient in our series, with a poor outcome, who did not show either 11q LOH or MYCN amplification, and this may have some significance in accounting for the relatively protracted course of the disease.

### Comparison of clinical features of tumours with MYCN amplification and 11q LOH

Chromosome 11q LOH and MYCN amplification clearly define 2 biologically distinct subgroups of aggressive neuroblastoma that appear to have different molecular aetiologies. It was of interest therefore to determine whether these subgroups also showed clinical differences. Table 2 shows the distribution of low- and high-stage disease, age and gender among patients in each of these genetic groups, as well as in the group of patients without genetic alteration. The most significant difference ($P$ = 0.015) observed was age at diagnosis (Table 2). All 10 patients with 11q LOH tumours were 1 year old or older, and 7 out of 10 were 3 years old or older. In contrast, the average age of patients in the MYCN amplified group was 1 year 6 months, with 4 out of 7 patients younger than 1 year old.

There were no significant differences in the MYCN amplified and LOH patient groups with respect to other clinical characteristics. However LOH tumours tended to occur at higher frequency in non-adrenal/abdominal sites (3 out of 10 tumours) as compared with MYCN-amplified tumours (0 out of 7 tumours), and also showed a very marked male predominance (9 out of 10 patients). These observations merit further investigation in a larger patient population.

### DISCUSSION

Accurate and reliable determination of probable prognosis is an important factor in the selection of the most appropriate treatment for children with neuroblastoma. In this study we have investigated the frequency and prognostic significance of LOH in four chromosomal regions that have previously been reported to be non-randomly deleted in neuroblastoma (3p, 4p, 11q and 14q). Our data represent the first detailed investigation in a single patient group, of the combined pattern of LOH on all 4 chromosomes and the relationship between LOH, MYCN amplification and chromosome 1p loss.

A moderate level of LOH (11–36%) was observed in each chromosomal region, with the highest frequency on chromosome 11.

**Table 2** Association between clinical and genetic characteristics of neuroblastoma patients

| | | | | Fisher's Exact Test for Association (*P*) | | |
|---|---|---|---|---|---|---|
| **Clinical features** | **MYCN amplification** | **11q LOH** | **No alteration*** | **All 3 groups** | **11q LOH vs no alteration** | **11q LOH vs MYCN amplification** |
| **All cases** | 7 | 10 | 11 | | | |
| **Tumour stage** | | | | **0.01** | **0.009** | 1.000 |
| 1, 2, 4S | 2 (29%) | 2 (**20**%) | 9 (**82**%) | | | |
| 3,4 | 5 (71%) | 8 (**80**%) | 2 (**18**%) | | | |
| **Age** | | | | **0.029** | 0.090 | **0.015** |
| < 1 year | 4 (**57**%) | 0 (**0**%) | 4 (36%) | | | |
| ≥ 1 year | 3 (**43**%) | 10 (**100**%) | 7 (64%) | | | |
| **Gender** | | | | 0.405 | 0.311 | 0.537 |
| Male | 5 (71%) | 9 (90%) | 7 (64%) | | | |
| Female | 2 (29%) | 1 (10%) | 4 (36%) | | | |

*Tumours without MYCN amplification and without 11q LOH.

 © 2001 Cancer Research Campaign

These results are in close agreement with frequencies reported by other workers (3p: 15%, Ejeskar et al, 1998; 4p: 23%, Caron et al, 1996; 11q: 32–44%, Srivatsan et al, 1993; Guo et al, 1999), suggesting that our study population, although relatively small, is representative of neuroblastoma patients generally. It is of interest however, that much higher levels of LOH on chromosome 14 reported in 3 Japanese studies (30–50%) (Hoshi et al, 2000; Suzuki et al, 1989; Takayama et al, 1992) are not evident in European/US populations (18–23%, Fong et al, 1992; Srivatsan et al, 1993; Theobald et al, 1999) and may reflect the contribution of tumours detected by mass screening. In the report of Hoshi et al (2000) for example, out of 17 neuroblastomas showing 14q LOH, 10 were detected by mass screening. These 10 tumours were all low stage in contrast to 6 out of 7 tumours not detected by screening, which were stage 3 or 4. This indicates that 14q alteration may be a relatively early change in the development of neuroblastomas, but that additional alterations are required for the acquisition of metastatic potential.

An unexpected finding in our study was a very high degree of concordance for LOH on chromosomes 3, 4, 11 and 14, strongly suggesting that this pattern of alteration is associated with a specific molecular pathway leading to neuroblastoma tumorigenesis. Furthermore, this pathway appears to be independent of MYCN amplification. This latter observation is in agreement with published reports noting an inverse correlation between MYCN amplification and LOH on chromosomes 4p (Caron et al, 1996), 11q (Guo et al, 1999) and 14q (Fong et al, 1992; Theobald et al, 1999). Further evidence in support of this suggestion is also provided by CGH studies. Vandesompele and co-workers for example noted a significant positive association between losses of 11q and 3p, or 11q and 14q, occuring predominantly in tumours without MYCN amplification (Vandesompele et al, 1998).

The requirement for multiple genetic alterations in the development of malignant tumours is widely accepted and a stepwise progression from premalignant to malignant stages is well illustrated in the case of colorectal cancer for example, where many of the alterations involved in the evolution of adenoma to carcinoma have been identified (Gryfe et al, 1997). The identification of premalignant lesions (neuroblastoma in situ) in fetal adrenal glands, together with a high frequency of clinically silent neuroblastomas among tumours detected by screening of infants, suggests that a similar process may occur in neuroblastoma. Although in general the majority of these premalignant tumours appear to be self-limiting, nevertheless in a small proportion, the deletion of genes on 14q for example, may provide an early step towards the acquisition of a more malignant phenotype. This is consistent with the observation that chromosome 14q loss is not predictive of survival (Fong et al, 1992; Takayama et al, 1992) despite the fact that it is often associated with advanced stage of disease. In contrast, a significant association between 11q loss and survival (Guo et al, 1999), suggests that this is a later event and may be associated with increased invasive and metastatic potential.

The results obtained in this study also show a very significant association between 11q loss and poor outcome. (In at least 8 of the 10 tumours showing LOH, cytogenetic,CGH and 11p genotyping data confirmed that loss was specific to 11q and was not a result of copy number abnormalities of the entire chromosome 11 (results not shown)). The 2-year overall survival rate was only 45%, and was similar to that seen in the MYCN-amplified group (51%), as compared with 100% survival in patients with single-copy MYCN, without 11q loss. The similarity in survival rate between MYCN and 11q LOH groups is consistent with the report of Lastowska et al (2000). In a study of 80 tumours in patients in the UK, Lastowska et al found that a distinct subgroup characterized by absence of MYCN amplification and frequent deletion of 11q, 1p, 3p and 4p had a 3-year survival rate of only 28% as compared with 24% for MYCN-amplified tumours and 100% for others. These results are in contrast to the findings of Guo et al (1999, 2000), who reported that 11q LOH was associated with an intermediate level of survival (2-year survival rate was 93%, 75% and 57%, for MYCN/11qLOH –/–, –/+ and +/– groups, respectively; Guo et al, 2000). There are a number of possible reasons for this difference. It may be significant that patients in the UK with neuroblastomas tend to be diagnosed at an older age and with higher stage disease than in other European countries and the USA (Powell et al, 1998) and might therefore be expected to show a poorer outcome. Patients with stage 4 disease make up approximately 60% of the total in the UK compared with approximately 40% elsewhere; the frequency of incidentally detected tumours is also lower in the UK (Powell et al, 1998). Differences in European and US treatment protocols may also have an effect on outcome. All of these factors emphasize that the value of prognostic markers is highly dependent on the characteristics of the patient population to which they are applied and therefore must be tested independently in each population.

The observed differences in survival associated with 11q LOH also allow some speculation about the characteristics of different tumour subgroups. It is possible that 11q tumours show a more progressive pattern of growth, with a longer lag period before clinical symptoms become apparent. Thus 11q LOH tumours, if detected at a relatively early stage of development, may have a better prognosis if treated appropriately. In contrast, MYCN amplification may produce an intrinsically more aggressive (and also more chemo-resistant) tumour that develops very rapidly and therefore is less likely to be detected prior to metastasis. These tumours have a uniformly poor prognosis in all populations studied.

It is important to consider the relationship between 11q loss and other molecular markers that have been shown to have prognostic significance in neuroblastomas. The most important of these are the loss of distal chromosome 1p and gain of 17q. In the case of chromosome 17q gain, it is of interest that this indicator of adverse outcome is observed in approximately 50% of both MYCN-amplified and non-amplified tumours (Bown et al, 1999). It should be noted however, that 17q gain very frequently arises as a result of unbalanced chromosome translocation with loss of material from the partner chromosome. Significantly, in MYCN-amplified tumours, t(1;17) is a very frequent finding, while in non-amplified tumours, chromosomes 3 or 11 may be involved in translocation with chromosome 17. We have noted for example, that early passage cell lines established from 2 of the 11q LOH patients described in this study showed t(11;17) (patient 28) or both t(3;17) and t(11;17) (patient 17), while the MYCN-amplified tumour of patient 22 showed t(1;17). We conclude therefore that the prognostic significance of chromosome 17q gain may be related at least in part to the specific partner chromosome involved in the unbalanced chromosome 17 translocation.

Although a close association between MYCN amplification and 1p36 loss is well established (Fong et al, 1989), the significance of 1p loss as an independent prognostic factor has been controversial (Gehring et al, 1995; Maris et al, 1995). It is now becoming clear however, that several different regions of loss are implicated and

*© 2001 Cancer Research Campaign* 

may be associated with different outcomes: small very distal deletions (1p36.2–36.3) are most frequently observed in patients with a good prognosis; larger deletions including the same region, but extending at least to 1p36.1 are associated with MYCN amplification, and a further more distal region of deletion at 1p22 is observed mostly in advanced-stage tumours (with and without MYCN amplification) (Schleiermacher et al, 1994; Takeda et al, 1994; Caron et al, 1995; Cheng et al, 1995; Schleiermacher et al, 1996; Mora et al, 2000). In this study 1p deletion was observed only in advanced-stage tumours, both with and without MYCN amplification, with a trend towards more extensive deletion (1p36 to at least 1p22) in the latter (Genus et al, unpublished). A 1p deletion was associated with a poor outcome in most cases. However of 13 patients with progressive/fatal disease, only 6 showed 1p deletion, whereas 12 out of 13 showed either 11q LOH or MYCN amplification. We conclude therefore that 1p loss is a less useful prognostic marker and that further investigation is required to allow more precise definition of the critical region(s) of deletion and the role played by each in tumorigenesis.

In conclusion, our results are consistent with a proposed classification of neuroblastomas into 3 distinct groups as outlined previously by Brodeur (1995), and in addition also provide a clear molecular basis for this classification. Thus, group 1 comprises low-stage tumours without MYCN amplification or other significant structural abnormalities, which have a very favourable prognosis. Group 2 tumours show 11q LOH (frequently accompanied by LOH on chromosomes 3, 4 and/or 14), are frequently stage 3 or 4, and lack MYCN amplification. The prognosis in these cases may be intermediate or poor. Group 3 tumours are characterized by MYCN amplification, advanced stage and an extremely poor prognosis. This system of classification has a number of important clinical implications; these include the possibility to identify at diagnosis most, if not all tumours with the potential for progressive metastatic growth, and those that require immediate and aggressive treatment. In addition the ability to distinguish between the majority of stage 1 and 2 tumours with a low risk of relapse, and a small but significant minority which are at high risk of relapse is an important factor in improving overall survival rates. Finally, the possibility that MYCN-amplified and 11q LOH tumours have distinct aetiologies and clinical behaviours suggests that each group should be considered separately in the optimization of treatment protocols, and the assessment of clinical trial results.

### ACKNOWLEDGEMENTS

This work was supported by grants from the Children Nationwide Medical Research Fund and the United Birmingham Hospitals Endowment Fund. We are grateful to the West Midlands Regional Clinical Genetics Service for data on MYCN amplification. We also thank Mrs S. Parkes of the West Midlands Regional Children's Tumour Registry, Birmingham Children's Hospital for access to patient clinical data.

### REFERENCES

Bown N, Cotterill S, Lastowska M, O'Neill S, Pearson ADJ, Plantaz D, Meddeb M, Danglot G, Brinkschmidt C, Christiansen H, Laureys G and Speleman F (1999) Gain of chromosome arm 17q and adverse outcome in patients with neuroblastoma. *N Eng J Med* **340**: 1954–1961

Brinkschmidt C, Poremba C, Christiansen H, Simon R, Schafer KL, Terpe HJ, Lampert F, Boecker W and Dockhorn-Dworniczak B (1998) Comparative genomic hybridization and telomerase activity analysis identify two biologically different groups of 4s neuroblastomas. Br J Cancer **77***: 2223–2229*

Brodeur GM (1995) Molecular Basis For Heterogeneity in Human Neuroblastomas. *Eur J Cancer* **31A**: 505–510

Brodeur GM, Maris JM, Yamashiro DJ, Hogarty MD and White PS (1997) Biology and genetics of human neuroblastomas. *J Ped Hematol/Oncol* **19**: 93–101

Brodeur GM, Pritchard J, Berthold F, Carlsen NLT, Castel V, Castleberry RP, de Bernardi B, Evans AE, Favrot M, Hedborg F, Kaneko M, Kemshead J, Lampert F, Lee REJ, Look AT, Pearson ADJ, Philip T, Roald B, Sawada T, Seeger RC, Tsuchida Y and Voute PA (1993) Revisions of the international criteria for neuroblastoma diagnosis, staging and response to treatment. *J Clin Oncol* **11**: 1466–1477

Caron H (1995) Allelic loss of chromosome 1 and additional chromosome 17 material are both unfavourable prognostic markers in neuroblastoma. *Med Pediatr Oncol* **24**: 215–221

Caron H, Peter M, van Sluis P, Speleman F, de Kraker J, Laureys G, Michon J, Brugieres L, Voute PA, Westerveld A, Slater R, Delattre O and Versteeg R (1995) Evidence for two tumour suppressor loci on chromosomal bands 1 p35–36 involved in neuroblastoma: one probably imprinted, another associated with N-myc amplification. *Hum Mol Genet* **4**: 535–539

Caron H, vanSluis P, Buschman R, doTanque RP, Maes P, Beks L, deKraker J, Voute P, Vergnaud G, Westerveld A, Slater R and Versteeg R (1996) Allelic loss of the short arm of chromosome 4 in neuroblastoma suggests a novel tumour suppressor gene locus. *Hum Genet* **97**: 834–837

Chalifa-Caspi V, Prilusky J and Lancet D (1998) The Unified Database. Weizmann Institute of Science, Bionformatics Unit and Genome Center (Rehovot, Israel): http://bioinformatics.weizmann.ac.il/udb

Cheng NC, VanRoy N, Chan A, Beitsma M, Westerveld A, Speleman F and Versteeg R (1995) Deletion mapping in neuroblastoma cell-lines suggests 2 distinct tumor-suppressor genes in the 1p35–36-region, only one of which is associated with N-myc amplification. *Oncogene* **10**: 291–297

Ejeskar K, Aburatani H, Abrahamsson J, Kogner P and Martinsson (1998) Loss of heterozygosity of 3p markers in neuroblastoma tumours implicate a tumour-suppressor locus distal to the FHIT gene. *Br J Cancer* **77**: 1787–1791

Fong C-T, Dracopoli NC, White PS, Merrill PT, Griffith RC, Housman DE and Brodeur GM (1989) Loss of heterozygosity for the short arm of chromosome 1 in human neuroblastomas: Correlation with N-myc amplification. *Proc Natl Acad Sci USA* **86**: 3753–3757

Fong C-T, White PS, Peterson K, Sapienza C, Cavenee WK, Kern SE, Vogelstein B, Cantor AB, Look AT and Brodeur GM (1992) Loss of heterozygosity for chromosome 1 or 14 defines subsets of advanced stage neuroblastomas. *Cancer Res* **52**: 1780–1785

Gehring M, Berthold F, Edler L, Schwab M and Ambler LC (1995) The 1p deletion is not a reliable marker for the prognosis of patients with neuroblastoma. *Cancer Res* **55**: 5366–5369

Gryfe R, Swallow C, Bapat B, Redston M, Gallinger S and Couture J (1997) Molecular biology of colorectal cancer. *Curr Probl Cancer* **21**: 238–301

Guo C, White PS, Weiss MJ, Hogarty MD, Thompson PM, Stram DO, Gerbing R, Matthay KK, Seeger RC, Brodeur GM and Maris JM (1999) Allelic deletion at 11q23 is common in MYCN single copy neuroblastomas. *Oncogene* **18**: 4948–4957

Guo C, White PS, Hogarty MD, Brodeur GM, Gerbing R, Stram DO and Maris JM (2000) Deletion of 11q23 is a frequent event in the evolution of MYCN single copy high risk neuroblastomas. *Med Ped Oncol* **35**: 544–546

Hoshi M, Otagiri N, Shiwaku H, Asakawa S, Shumizu N, Kaneko Y, Ohi R, Hayashi Y and Horii A (2000) Detailed deletion mapping of chromosome band 14q32 in human neuroblastoma defines a 1.1 Mb region of common allelic loss. *Br J Cancer* **82**: 1801–1807

Kerbl R, Urban CE, Lackner H, Hofler G, Ambros IM, Ratschek M and Ambros PF (1996) Connatal localized neuroblastoma. *Cancer* **77**: 1395–1401

Lastowska M, Nacheva E, McGuckin A, Curtis A, Grace C, Pearson A and Bown N (1997) Comparative genomic hybridization study of primary neuroblastoma tumours. *Genes Chromosomes Cancer* **18**: 162–169

Lastowska M, Cullinane C, Variend S, Cotterill S, Bown N, O'Neill S, Mazzocco K, Roberts P, Nicholson J, Ellershaw C, Pearson A and Jackson M (2000) Genetic and histopathological analysis of neuroblastoma tumours. *Med Ped Oncol* **35**: 204

Maris J, White P, Beltinger C, Sulman E, Castleberry R, Shuster J, Look A and Brodeur G (1995) Significance of chromosome 1p loss of heterozygosity in neuroblastoma. *Cancer Res* **55**: 4664–4669

Mora J, Cheung N, Kushner B, LaQuaglia M, Kramer K, Fazzari M, Heller G, LS C and Gerald W (2000) Clinical categories of neuroblastoma are associated with different patterns of loss of heterozygosity on chromosome are 1p. *J Mol Diag* **2**: 37–46

Plantaz D, Mohapatra G, Matthay KK, Pellarin M, Seeger RC and Feuerstein BG

 © 2001 Cancer Research Campaign

(1997) Gain of chromosome 17 is the most frequent abnormality detected in neuroblastoma by comparative genomic hybridization. *Am J Pathol* **150**: 81–89

Powell JE, Esteve J, Mann JR, Parker L, Frappaz D, Michaelis J, Kerbl R, Mutz ID and Stiller CA (1998) Neuroblastoma in Europe: differences in the pattern of disease in the UK. *Lancet* **352**: 682–687

Schleiermacher G, Delattre O, Peter M, Mosseri V, Delonlay P, Vielh P, Thomas G, Zucker JM, Magdelenat H and Michon J (1996) Clinical relevance of loss of heterozygosity of the short arm of chromosome 1 in neuroblastoma: a single institution study. *Int J Cancer* **69**: 73–78

Schleiermacher G, Peter M, Michon J, Hugot JP, Vielh P, Zucker JM, Magdelenat H, Thomas G and Delattre O (1994) Two distinct deleted regions on the short arm of chromosome 1 in neuroblastoma. *Genes Chromosomes Cancer* **10**: 275–281

Srivatsan ES, Ying KL and Seeger RC (1993) Deletion of chromosome 11 and 14q sequences in neuroblastoma. *Genes Chromosomes Cancer* **7**: 32–37

Suzuki T, Yokota J, Mugishima H, Okabe I, Ookuni M, Sugimura T and Terada M (1989) Frequent loss of heterozygosity on chromosome 14q in neuroblastoma. *Cancer Res* **49**: 1095–1098

Takayama H, Suzuki T, Mugishima H, Fujisawa T, Ookuni M, Schwab M, Gehring M, Nakamura Y, Sugimura T, Terada M and Yokota J (1992) Deletion mapping of chromosome-14q and chromosome-1p in human neuroblastoma. *Oncogene* **7**: 1185–1189

Takeda O, Homma C, Maseli N, Sakurai M, Kanda N, Schwab M, Nakamura Y and Kaneko Y (1994) There may be two tumor suppressor genes on chromosome arm 1p closely associated with biologically distinct subtypes of neuroblastoma. *Genes chromosome Cancer* **10**: 30–39

Thompson PM, Kyemba SK, Jensen SJ, Guo C, Maris JM, Brodeur GM, Stram DO, Matthay KK, Seger RC and White PS (2001) Loss of heterozygosity (LOH) from chromosome 14q in neuroblastoma. *Med Ped Oncol* (in press)

Theobald M, Christiansen H, Schmidt A, Melekian B, Wolkewitz N, Christiansen NM, Brinkschmidt C, Berthold F and Lampert F (1999) Sublocalization of putative tumor suppressor gene loci on chromosome arm 14q in neuroblastoma. *Genes Chromosomes Cancer* **26**: 40–46

Vandesompele J, Van Roy N, Van Gele M, Laureys G, Ambros P, Heimann P, Devalck C, Schuuring E, Brock P, Otten J, Gyselinck J, De Paepe A and Speleman F (1998) Genetic heterogeneity of neuroblastoma studied by comparative genomic hybridization. *Genes Chromosomes Cancer* **23**: 141–152

*© 2001 Cancer Research Campaign*
